# Supplementary material for: Patterns of Intron Gain and Loss in Fungi
Source: PLoS Biol. 2004 Nov 30;2(12):e422. doi: 10.1371/journal.pbio.0020422 (PMC532390; doi:10.1371/journal.pbio.0020422)
Supplement: Table S1 — Also available at http://genes.mit.edu/NielsenEtAl/. (4.3 MB ZIP). [file pbio.0020422.st001.zip › NielsenEtAl/html/1254.html]

AN6898.1.NCU00435.1.MG05208.1.FG04112.1


```
 CLUSTAL W (1.82) Multiple Sequence Alignments - Introns Inserted


Sequence 1: NCU00435.1	227 aa
Sequence 2: MG05208.1	223 aa
Sequence 3: FG04112.1	228 aa
Sequence 4: AN6898.1	228 aa
Alignment Length: 253 aa
Number Identitical Residues: 131 aa
Alignment Score (without introns) 6066


MG05208.1 	MS~DAW0IIEWAFGKRMTPAERLRKNQRLLDKAIRELDQQRVKLEKQEKTLVTQIKQSAQ
NCU00435.1	MN0---~VLEWAFGKRMTPAERLRKNQRLLDKAIRELDQQRVKLEKQEKTLVSQIRQSAQ
FG04112.1 	MN0---~ILEWAFGKRMTPAERLRKNQRMLDKAIRELDQTRVKLEKQEKTLIQQIKTSAK
AN6898.1  	MN0---~IVEWAFGKRMTPAERLRKHQRALDRTQRELDRERTKLENQEKKLIQDIKKSAK
          	*.     ::****************:** **:: ****: *.***:***.*: :*: **:

MG05208.1 	KNQMGACKIQAKDL~VRTRRYIEKFYGMRSQLQQISLRLQ~THRTNEQMMQAMKGATMAL
NCU00435.1	KGQMGACKIQAKDL~VRTRRYIEKFYSMRSQLQKISLRLQ0TYRTNEQMMQAMKGATMAL
FG04112.1 	NGQMGACKIQAKDL~VRTRRYVEKFYSMRSQLQKISLRLQ0TYRTNEDMMQAMKGATRAL
AN6898.1  	NGQIGACKIQAKDL0A------------------ISLRIQ0TVRSNEQMMQSMKGATMLL
          	:.*:********** .                  ****:* * *:**:***:*****  *

MG05208.1 	GSMNRTMNLPSLQRIAAEFERENDIMEQRQELMDDAVDDAMDV-GVEEEGDELVEQVLEE
NCU00435.1	GSMNRTMNLPSLQRIAMEFERENDIMEQRQEMMDDAIDDAMDM-GLEEEGDEVVEQVLEE
FG04112.1 	GSMNKQMNLPQLQRIAMEFERENDIMDQRSEVMDDVFDDAMDV-GVEEEGDEVVEQVLEE
AN6898.1  	GSMNRQMNLPALQRIAMEFERENDIMDQRQEMMDDAIDEATGIEGEEEEGEDIVKEVLDE
          	****: **** ***** *********:**.*:***..*:* .:.* ****:::*::**:*

MG05208.1 	IGVDLKNS0--------------------LGETPQGLQSETVAETRIAQAVGGGS-----
NCU00435.1	IGVDLSQA0--------------------MGETPSGLQVAAVPENKVAQAVGGGGGGGGA
FG04112.1 	IGVDFNQA0--------------------LGETPTALGTAAVPEGKIAQAVGGGGGGGGG
AN6898.1  	IGVDLSQA~VRILLLVYVDMDRTNASLKQLGETPSGIQKESVSEGRVAQAIGAGGAGGAS
          	****:.::          . . :.:: ..:**** .:   :*.* ::***:*.*......

MG05208.1 	----DDDLQARLDSLRPK
NCU00435.1	-DPGDDDLQARLDSLRR-
FG04112.1 	GDPVDDDLQARLDSLRK-
AN6898.1  	---DDDDLQARLDSLRR-
          	    ************
```
